# *Agrobacterium rhizogenes*-Mediated Transformation for Generation of Composite Sugar Beet with Transgenic Adventitious Roots

**DOI:** 10.3390/plants14172747

**Published:** 2025-09-02

**Authors:** Yue Sun, Yiduo Zhao, Minshi Jia, Xudong Zhang, Xixuan Zhou, Shengnan Li, Zedong Wu, Zhi Pi

**Affiliations:** 1 Academy of Modern Agriculture and Ecological Environment, Heilongjiang University, Harbin 150080, China; sy897751873@163.com (Y.S.); lishengnan@hlju.edu.cn (S.L.); 2 Key Laboratory of Sugar Beet Genetic Breeding, Heilongjiang University, Harbin 150080, China; 3 Engineering Research Center of Agricultural Microbiology Technology, Ministry of Education & Heilongjiang Provincial Key Laboratory of Ecological Restoration and Resource Utilization for Cold Region & Key Laboratory of Microbiology, College of Heilongjiang Province & School of Life Sciences, Heilongjiang University, Harbin 150080, China

**Keywords:** sugar beet, adventitious roots transformation, *Agrobacterium rhizogenes*, composite plants

## Abstract

Sugar beet (*Beta vulgaris* L.), a biennial sugar crop, provides about 16% of the world’s sucrose production. PEG and *Agrobacterium tumefaciens*-mediated transformation have been established for sugar beet. However, the traditional transformation of sugar beet is time-consuming, low efficiency, and dependent on tissue regeneration. Recently, the use of *Agrobacterium rhizogenes* for genetic transformation without tissue culture has become a new possibility. Here, we describe an optimized *A. rhizogenes*-mediated transformation for the generation of composite sugar beet without tissue culture. By dipping *A. rhizogenes* K599 colonies onto a wound of hypocotyl and petiole, about 81.7% and 51.1% of shoots and leaves could be induced to produce adventitious roots. Of these, more than 60% of the explants contained transformed adventitious roots. Specifically, we discovered that the transformation efficiency was significantly improved when the *MAS* promoter was employed instead of the *CaMV35S* promoter. The transformation in adventitious roots was also validated by qRT-PCR and Western blot at the transcriptional and translational levels. The transformed adventitious roots have great potential for the study of taproot development, sugar accumulation, and resistance to root diseases, which is closely related to sugar beet yield and quality.

## 1. Introduction

Plant transformation serves as an effective strategy to enhance yield, quality, and resistance to both abiotic and biotic stresses [1]. Given that the ability of *Agrobacterium* to naturally insert DNA into the host genome has been recognized for over 40 years, numerous important crops, such as maize (*Zea mays* L.), soybean (*Glycine max* L. Merr.), cotton (*Gossypium* spp.), canola (*Brassica napus* L.), potato (*Solanum tuberosum* L.), and tomato (*Solanum lycopersicum* L.), have been genetically transformed [2]. Typically, *Agrobacterium tumefaciens*-mediated transformation is often accomplished by infecting the callus, leaves, cotyledons, and other plant parts, followed by tissue culture for plant regeneration. However, the processes of transformation and regeneration are commonly time-consuming and highly dependent on the genotype [3]. This represents a serious challenge to the practical application of transgene and gene editing in molecular function research and molecular breeding. Beside *A. tumefaciens*-mediated transformation, *A. rhizogenes* is another class of bacteria capable of transferring exogenous genes into the host genome. As each adventitious root from a single cell represents an independent transformation event, *A. rhizogenes*-mediated transformation is a high-throughput way to induce transgenic hairy root lines quickly. It has been reported that more than 100 plant species, which mainly belong to Solanaceae, Asteraceae, Cruciferaceae, Convolvulaceae, Umbelliferae, Leguminosae, Caryophyllaceae, and Polygonaceae, can be induced to produce adventitious roots through *A. rhizogenes* infection [4]. The transgenic events could be detected in the adventitious roots of soybean and cucumber (*Cucumis sativus* L.) simply by dipping colonies or immersing the solution into wounds [5,6]. Recently, Cao et al. [7] presented an extremely simple *A. rhizogenes*-mediated transformation method, namely the cut–dip–budding (CDB) delivery system. A number of dicots and monocots, including two herbaceous plants, a tuberous root plant (sweet potato), and three woody plant species, successfully achieved heritable transformation by CDB. Additionally, the modified CDB can also be used to transform succulent plants, which possess the capacity to regenerate from leaves [8]. Transformation efficiencies of around 74% and 5% are attained for *Kalanchoe blossfeldiana* and *Crassula arborescens* by dipping and coating *Agrobacterium* onto hypertrophic leaves. By substituting dipping with vacuum infection, CDB was also applicable to the transformation of the monocotyledonous *Sansevieria trifasciata*. Transformation efficiencies ranging from 3.9% to 7.8% were achieved in different genotypes. These studies highlight the remarkable advantages of *A. rhizogenes*-mediated transformation in terms of its wide species applicability, simplicity, and efficiency.

Besides *Agrobacterium* species, the selection of promoter is also a crucial factor to consider for the transformation [9]. The *Cauliflower mosaic virus 35S* (*CaMV35S*) promoter, as a constitutive promoter of non-plant origin, is widely employed in various applications, ranging from fundamental research in plant biology to the development of commercial crop varieties with new agriculturally relevant traits, such as insect resistance and herbicide tolerance [10]. Currently, for over 60% of transgenic crops, including soybeans, cucumbers, and tomatoes, the *CaMV35S* promoter is chosen for the stable expression of exogenous genes [10]. Although the *CaMV35S* promoter and its derivatives can drive strong expression of target genes in dicotyledonous plants, their activities are significantly lower in monocotyledonous plants [11]. Among dicotyledonous plants, emerging evidence suggests that selective utilization of the other constitutive promoters may lead to superior outcomes in genetic transformation and gene editing, compared to the conventional *CaMV35S* promoter. The promoter of the *mannopine synthase* (*MAS*) gene has been isolated from *Agrobacterium* and inserted into the upstream of the desired gene in transformation vectors. In pyrethrum *GUS* transgenic plants, both the number of GUS spots per segment and GUS activity were roughly 30% higher in plants utilizing the *MAS* promoter than in those using the *CaMV35S* promoter [12].

Sugar beet (*Beta vulgaris* L.), a biennial sugar crop, stores approximately 16 to 18% sugar in its taproots and contributes about 16% of the world’s sucrose production. At the close of the last century, the *uidA* gene was successfully introduced into the sugar beet genome through PEG-mediated transformation of guard cell protoplasts [13]. The transgenic plants exhibit resistance to glufosinate-ammonium-based herbicides and are suitable for breeding purposes. In recent years, numerous studies have reported using *Agrobacterium* to infect leaves, cotyledons, and shoots for the regeneration of transgenic plants [14,15]. Factors including strain, bacterial solution concentration, hormone species, and hormone concentrations are also systematically optimized for higher transformation efficiency [16]. However, these transformation methods typically are time-consuming, highly reliant on aseptic procedures, and difficult to replicate without minor adjustments across different laboratories. Because naked protoplasts are extremely fragile and sensitive, the optimization of electric pulse fields is often required for electroporative transformation [17]. For PEG-mediated transformation, the concentration requirements for each component in the resuspension solution are highly stringent. Even the use of reagents from different brands can result in the fragmentation of protoplasts. Vitrification and poor plantlet establishment during tissue regeneration frequently result in the failure of regenerating transformed plants [17]. Composite plants are inherently chimeric, with transgenic roots and wild-type shoots. When studies focus on genes involved in root biology, composite plants induced by *A. rhizogenes*-mediated transformation provide a time-efficient alternative to the typically laborious and ineffective stable genetic transformation methods [18]. Large-scale gene characterization studies can be carried out efficiently due to the reduction in plant production costs. Composite plants have been utilized to explore traits such as symbiosis and root pathogen interactions, root growth and development, and abiotic stresses [19,20]. Hence, composite plants have a potential for studying taproot development, sugar accumulation, and resistance to root diseases, which was closely related to sugar beet yield and quality. In this study, we describe a method for the induction of transgenic composite plants in sugar beet under non-sterile conditions using *A. rhizogenes*. It expands the range of approaches for functional genetics in sugar beet, enabling the characterization of candidate genes associated with root development and stress adaptation.

## 2. Results

### 2.1. Effect of A. rhizogenes on Adventitious Root Induction from Sugar Beet Shoots and Leaves

**Figure 1:**
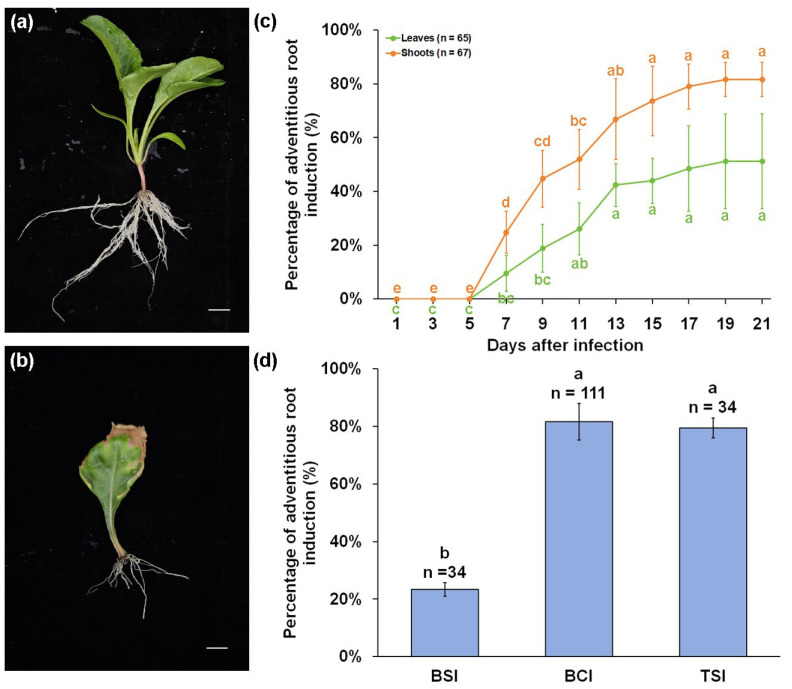
The induction of adventitious roots from shoots and leaves of sugar beet by *A. rhizogenes* K599. (**a**) Induced hairy roots from shoots at 21 days after infection. (**b**) Induced hairy roots from leaves at 21 days after infection. (**c**) The increase in percentage of adventitious roots within 21 days after BCI. (**d**) The effects of three infection strategies on adventitious root induction. BSI, BCI, and TSI represent three infection strategies, namely bacterial suspensions infection, bacterial colony infection, and two-step infection. Scale bars = 1 cm. Error bars represent SD of three replicates. Different lowercase letters represent significant differences (*p* < 0.05).

To evaluate the efficacy of *A. rhizogenes* in inducing adventitious roots, shoots without roots and part of the hypocotyl were harvested from 10-day-old seedlings, while fully expanded leaves were collected from 14-day-old seedlings. The wound was successively immersed in *A. rhizogenes* K599 suspension and coated with bacterial colony (Figure S1). The infected shoots and leaves were transplanted in a plastic box with a lid to maintain moisture. After 3 weeks of growth, adventitious roots were observed to be induced from the wounds of hypocotyls and petioles (Figure 1a,b). Induction from shoots was superior to that from leaves for both the number and viability of induced adventitious roots. Approximately 74 adventitious roots could be induced from each shoot. By contrast, only around 13 adventitious roots could be induced from the petiole. The length of adventitious roots induced from shoots were about 4.64 cm, which was about 2.1 times longer than those induced from leaves. In addition, the efficiency of adventitious roots induction from shoots was significantly higher than that from leaves. The percentage of adventitious roots induced in shoots and leaves reached 81.7% and 51.1% at 3 weeks after infection (Figure 1c). The induction of adventitious roots could be divided into three stages over a 3-week growth period. The adaptation stage of the explants occurred within 5 days after infection. The leaves growing after infection wilted easily and showed signs of necrosis, due to the absence of roots in the explants. The root primordium emergence could be observed 3 days after infection. Approximately 5 to 10% of the explants might die at this stage, depending on the health of the explants and environmental humidity. The percentage of adventitious roots induction in shoots and leaves were 24.8% and 9.5%, respectively. More shoots and leaves were stimulated to generate adventitious roots between 7 and 13 days after infection. From 13 to 21 days after infection, the explants resumed normal shoot growth, but the rate of induction of adventitious roots did not change significantly. We also compared the effects of the three infection strategies, including bacterial suspensions infection (BSI), bacterial colony infection (BCI), and two-step infection (TSI), on the percentage of adventitious root induction (Figure 1d). About 23.3% shoots were successfully induced adventitious roots as a result of BSI. However, this percentage of adventitious root induction was significantly lower than those using BCI and TSI. The percentage of adventitious root induction using BCI and TSI were 81.7% and 79.4%, respectively. There was no significant change between BCI and TSI. BCI was chosen for subsequent experiments because of its high efficiency in inducing adventitious roots and the simplicity of the operation.

### 2.2. Transformation Efficiency of Adventitious Roots in Sugar Beet

**Figure 2:**
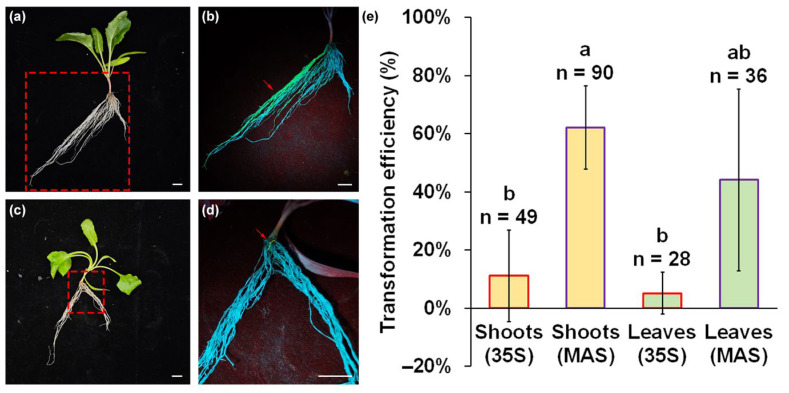
The transformation efficiency of adventitious roots induced from shoots and leaves. (**a**) Adventitious roots induced from shoots using plasmids containing *MAS* promoter. (**b**) The close-up of eGFP fluorescence observation at the box marker in section (**a**). (**c**) Adventitious roots induced from shoots using plasmids containing *CaMV35S* promoter. (**d**) The close-up of eGFP fluorescence observation at the box marker in section (**c**). (**e**) Transformation efficiency under different infection sites and expression vectors. Scale bars = 1 cm. Error bars represent SD of three replicates. The red arrow in (**b**,**d**) indicates the adventitious roots with eGFP signal that have been transformed. Different lowercase letters represent significant differences (*p* < 0.05).

To assess the transformation efficiency using *A. rhizogenes* K599, we constructed eGFP expression plasmids with *CaMV35S* and *MAS* promoter and inspected GFP fluorescence signals in adventitious roots under a LUYOR-3415 light source. Following the emergence of adventitious root primordia at 3 days after infection, the adventitious roots elongated to over 1 mm and exhibited a visible eGFP fluorescence at 7 days after infection. The adventitious roots induced by *A. rhizogenes* K599 without the eGFP expression plasmid exhibited a grayish-white color, whereas the transgenic adventitious roots induced by *A. rhizogenes* K599 with the eGFP expression plasmid turned bright green under the illumination of light source at 440 nm (Figure S3). About 40% of adventitious roots displayed a visible green fluorescence signal upon employing an expression plasmid utilizing *MAS* promoter at 21 days after infection (Figure 2a,b). The fluorescence signal was found in all parts of the adventitious roots. However, only a weak green fluorescence signal could be detected in the root tips and a few young roots, when the eGFP expression plasmid with the *CaMV35S* promoter was used for transformation (Figure 2c,d). Among seedlings, from the induction of adventitious roots from shoots, approximately 62.1% of the seedlings were observed to have undergone successful transformation after infection by *A. rhizogenes* K599 containing the eGFP expression plasmid with the *MAS* promoter (Figure 2e). The induction of adventitious roots from leaves still maintained about 44.1% transformation efficiency. However, there was only 11.1% and 5.1% transformation efficiency when infection was performed using *A. rhizogenes* K599 containing the eGFP expression plasmid with the *CaMV35S* promoter. Considering the percentage of adventitious root induction, transformation efficiency, and eGFP signal strength, only shoots infected with the eGFP plasmid with *MAS* promoter were used for subsequent experiments.

### 2.3. The Long-Term Stability and Spatial Distribution of eGFP Expression in Adventitious Roots

**Figure 3:**
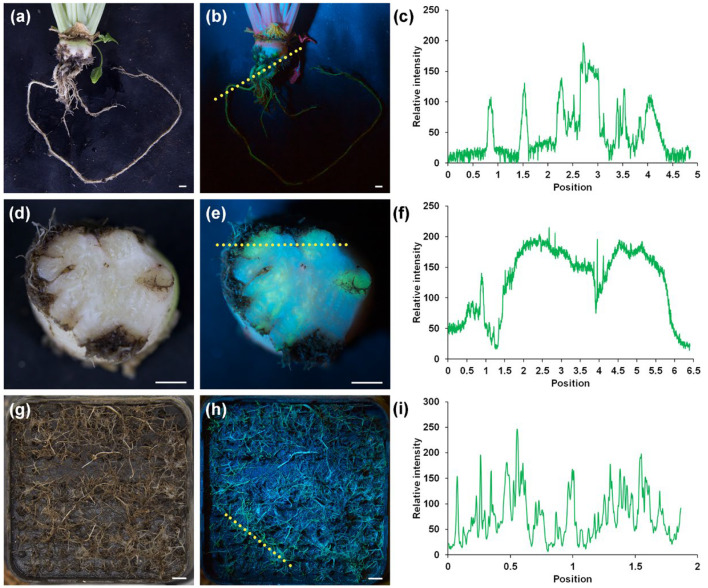
The eGFP fluorescence observed in 3-month-old sugar beets. (**a**) The morphology of composite plant after 3-month growth. (**b**) eGFP fluorescence observed in coarse roots. (**c**) The relative intensity of eGFP changes from left to right in the line labeled in (**b**). (**d**) Transverse section at the root neck. (**e**) eGFP fluorescence observed in root neck section. (**f**) The relative intensity of eGFP changes from left to right in the line labeled in (**e**). (**g**) Numerous top tips outside the bottom of pot. (**h**) Numerous top tips have eGFP fluorescence. (**i**) The relative intensity of eGFP changes from left to right in the line labeled in (**h**). Scale bars = 1 cm. A relative intensity exceeding 100 indicates visible eGFP expression.

To observe the growth and development of adventitious roots and the expression of eGFP over a long-term period, positively transformed seedlings and seedlings infected by *A. rhizogenes* K599 without plasmid were cultivated in the cultivation room for 3 months. In contrast to the adventitious roots induced by *A. rhizogenes* K599 without the eGFP expression plasmid, distinct eGFP fluorescence signals were detected both at the infection site of the root neck and in the thickened adventitious roots of *A. rhizogenes* K599 (Figure S4). To relatively quantify the fluorescence signal intensity of eGFP, we utilized the plot profile function of Image J to analyze the captured eGFP expression images. In the root without eGFP expression, the relative intensity ranged from 0 to 50. In contrast, the relative intensity of the regions with visible eGFP expression exceeded 100. These coarse roots, whose diameter increased to approximately 0.8 cm, exhibited a distinct eGFP signal (Figure 3a,b). The relative intensity of this signal ranged from 104.4 to 246.7. (Figure 3c). Unexcised hypocotyls were also enlarged to form the root neck (Figure 3d). In transverse sections of the root neck, about 30% of the area presented an obvious eGFP fluorescence signal (Figure 3e). These regions, with the strongest relative fluorescence intensity reaching 215 (Figure 3f), were mainly distributed at the position of transformed coarse roots and the area adjacent to the site of infection with *A. rhizogenes*. These cells might have originated from cells that were transformed by *A. rhizogenes* in the hypocotyl. Owing to the small cell size and dense cellular arrangement within the meristem, the root tip emerges as another site where eGFP fluorescence can be distinctly seen (Figure 3g,h). Stable expression of eGFP was found in over 70% of the root tips and the mean relative fluorescence intensity reached 136.2 (Figure 3i). These results supported the idea that *A. rhizogenes* could introduce the exogenous *eGFP* gene into adventitious roots for long-term stable expression.

### 2.4. The Validation of Transformation in Adventitious Roots at Molecular Level

**Figure 4:**
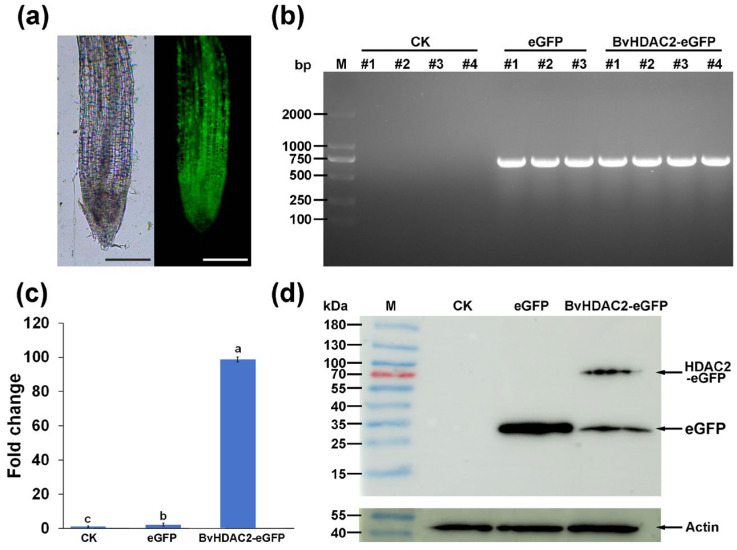
The validation of transformation in adventitious roots at the transcriptional and translational level. (**a**) The observation of eGFP fluorescence in root tips by fluorescence microscope. Scale bars = 0.5 mm. (**b**) The PCR amplification of *HPT* gene in the root before and after transformation. (**c**) The up-regulation of *BvHDAC2* in adventitious roots. The roots harvested from wild-type sugar beet designated as the control (CK). Error bars represent SD of three replicates. Different lowercase letters represent significant differences (*p* < 0.05). (**d**) The overexpression of *BvHDAC2* detected by Western blot.

To confirm the effect of *A. rhizogenes*-mediated transformation on the overexpression of exogenous genes in sugar beet, we constructed a BvHDAC2-eGFP fusion protein expression vector for adventitious root transformation. Except for at the root cap, eGFP signals were detected in all zones of the root tip (Figure 4a). PCR amplification with cDNA extracted from the root before and after transformation indicated that the transcriptional expression of the *HPT* gene, which is a resistance gene located between the RB and LB regions, could only be detected in the transformed adventitious roots (Figure 4b). In addition, we employed qRT-PCR to measure the relative transcriptional expression of *BvHDAC2* among wild type and seedlings transformed with eGFP and the BvHDAC2-eGFP fusion protein expression vector. After the overexpression of *BvHDAC2* by *A. rhizogenes*-mediated transformation, the transcriptional abundance of *BvHDAC2* was more than 98.7-fold and 43.9-fold higher compared to that of wild-type plants and those transformed with eGFP expression vector, respectively (Figure 4c). At the protein level, the specific band of the eGFP was located at about 27 kDa, which was consistent with the expected molecular weight. For seedlings transformed with BvHDAC2-eGFP fusion protein expression vector, another specific band located at about 76 kDa was detected with a molecular weight consistent with the sum of the molecular weights of BvHDAC (39.1 kDa) and eGFP (26.9 kDa). These results confirmed the expression of the BvHDAC2-eGFP fusion protein in the transformed adventitious roots (Figure 4d).

## 3. Discussion

In sugar beet, various transformation techniques, including *Agrobacterium*-mediated, electroporation, PEG-mediated, and sonication methods, have been utilized for gene transfer [17]. Although genetic transformation has led to an increase in sugar yield, disease resistance, and herbicide resistance in some instances, sugar beet is still regarded as recalcitrant to genetic transformation [20]. Sugar beet researchers frequently face the issue that the transformation methods reported by other laboratories cannot be reproduced in another laboratory. The reasons for these problems include the genotype-dependence of the methods, low transformation efficiency requiring an extremely large number of explants, and the complexity of multi-step protocols.

Owing to genotype dependency, the published transformation protocols frequently employ markedly divergent combinations of plant growth regulator types and concentrations. Moreover, breeding materials, such as the cytoplasmic male-sterile (CMS) line, Owen-type maintainer line, and pollination line, are typically used in previous methods [14,15,16]. It is often extremely challenging for another laboratory to acquire these materials, due to the influence of proprietary protection of breeding materials and national policies regarding the import and export of germplasm resources. Once an attempt is made to replace the material for genetic transformation, it usually requires long-term and systematic re-optimization. In our study, KWS9147, a common commercial variety that can be easily purchased in various regions, was chosen for genetic transformation. This ensures the reproducibility of our method in different laboratories. Among sunflower and Chinese cabbages, adventitious root transformation mediated by *A. rhizogenes* has been reported as an alternative to overcome genotype-dependent limitations [21,22]. Although we have not systematically calculated the transformation efficiency of adventitious roots in different sugar beet varieties, we discovered that the transformation of adventitious roots by *A. rhizogenes* was also effective in four sugar beet Owen-type maintainer lines during the preliminary experiments. This suggests that *A. rhizogenes* may exhibit low genotype dependence during the transformation of sugar beet adventitious roots.

Another bottleneck of the existing transformation method is low transformation efficiency. Regardless of the explants such as shoot bases, petioles, leaves, callus, and cotyledons, the induction rate of adventitious buds is generally no more than 10% and transformation efficiency of sugar beet was merely between 0.1% and 1% [23]. Low transformation efficiency led to the requirement of an extremely large number of explants, causing a significant obstacle for the genetic analysis of sugar beet. For *A. rhizogenes*-mediated transformation, the induction rate of adventitious roots is typically more than 80% in some species, including soybean, cucumber, and sunflowers [5,6,21]. In previous sonication-assisted *A. rhizogenes*-mediated transformation experiments, approximately 30% of the plants developed adventitious roots [24]. The transformation efficiency was 54.8%, meaning that only 16.4% of the explants could successfully induce transgenic adventitious roots [24]. In our new method, the efficiency of adventitious root induction and transformation also showed a significant increase. The percentage of adventitious root induction was able to exceed 80%, and the transformation efficiency of seedlings with induced adventitious roots was able to reach up to 62.1%. The efficiency of inducing transgenic adventitious roots from explants was approximately 33% higher than that of previous sonication-assisted *A. rhizogenes*-mediated transformation. The high transformation efficiency enables research on the functions of batch genes, such as gene families and candidate genes screened by various omics.

Regardless of whether it is *A. tumefaciens*-mediated, electroporation, PEG-mediated, or the sonication method, an appropriate regeneration system is always required for the development and improvement of stable transgenics [25]. The regeneration system commonly consists of explant sterilization, preincubation, co-culture, adventitious bud induction, and adventitious root induction. This multi-step process makes optimization complex and time-consuming. Meanwhile, improper hormone use or bacterial contamination at each step can lead to the failure of transformation. The previous protocol for sonication-assisted *A. rhizogenes*-mediated transformation in sugar beet still required tissue culture [24]. In contrast, the induction of transformed adventitious roots could be achieved simply by dipping the wound on bacterial colonies and co-culturing it with appropriate moisturization, as in our new method. Complicated operations such as ultrasonic-assisted infection, NAA induction, and sterile culture were not necessary. Consequently, with the use of our novel method, researchers, even those without prior tissue culture experience, can easily obtain sufficient quantities of transgenic adventitious roots for root studies in sugar beet.

We also discovered that there are significant differences in transformation efficiencies on adventitious roots in sugar beet among different constitutive promoters. The *CaMV35S* promoter is one of the most widely used for overexpression of foreign genes. In sugar beet, *CaMV35S* was also frequently selected as the promoter for the overexpression of target genes. Although the use of the *CaMV35S* promoter resulted in the expression of eGFP in adventitious roots in this study, the fluorescence signal from eGFP expression was quite weak. We only observed green fluorescence in the apical region, which might be ascribed to the tight cell density and dense cytoplasm in the apical meristem. In contrast, the fluorescence visible region expanded from the root tip to the entire root, when the *MAS* promoter was used instead of the *CaMV35S* promoter. Based on previous studies, the *MAS* promoter could enhance the expression of β-glucuronidase activity by 2–20 times in tobacco and maize compared with the commonly utilized enhanced *CaMV35S* promoter [26,27]. It has also been reported that substituting the *CaMV35S* promoter with the *MAS* promoter can enhance the efficiency of gene editing by approximately 7.5% in poplar [28]. Generally, a constitutive promoter can activate the expression of genes all the time and anywhere in the plant. However, many studies suggested that genes regulated by a constitutive promoter are not always expressed in all tissue types [10]. A high percentage of *MAS* promoter activity commonly occurred in the auxin-rich hairy roots, owing to the consensus auxin-inducible promoter element [29]. The wound of the explant may also be a trigger for the induction of genes by the *MAS* promoter [29]. These might be the reasons for the better effect of *A. rhizogenes*-mediated transformation using the *MAS* promoter. In summary, we proposed that the utilization of the *MAS* promoter might be capable of achieving superior transgenic and gene editing effects in sugar beet.

So far, adventitious root transformation mediated by *A. rhizogenes* has been widely utilized for various studies on plant–pathogen interactions, stresses resistance, root nutrient uptake, and phytohormone transport [30,31]. In soybean and cucumber, the ability of the *YAO* promoter to drive the expression of downstream *GUS* gene expression was identified by observing GUS activity in the adventitious roots [5,6]. The characterization of the *GmPRP2* promoter was analyzed through GUS reporter assays in transgenic hair roots, and its core fragment for root-preferential expression was found to be between −369 and +1 [32]. These studies imply that activities, enhancers, repressors, and the core region of the root-specific promoter can be readily detected through *A. rhizogenes*-mediated transformation. Since fleshy taproot is an important organ for sugar beet harvest, breeding and gene function studies have traditionally focused on increasing the yield and sugar content of taproot [33]. The transformed adventitious roots induced by *A. rhizogenes* can be conveniently used to study the activity of promoters, transcription factors, and enzymes in the root. It also provides a new type of organ site for validating the alterations in root phenotype induced by the overexpressed gene in sugar beet. Recently, the interaction between the sugar beet roots and rhizosphere microorganisms has become an area of concern, which is closely associated with continuous cropping effects and root rot [34,35]. Owing to the advantages of rapidly and efficiently obtaining transgenic roots via *A. rhizogenes*-mediated transformation, field tests to assess the potential of transgenic plants to resist continuous cropping and root rot have become feasible.

In several plant species, such as sweet potato, crown vetch, and rubber dandelion, it has been feasible to obtain transgenic plants induced by the CDB delivery system [7]. The success of these cases has been ascribed to the ability of transgenic plants to regenerate stems and leaves from their roots, which is known as “root suckering”. Unfortunately, the taproot of sugar beet does not have the ability to regenerate shoots spontaneously, which limits the acquisition of transgenic plants by *A. rhizogenes*-mediated transformation. There are several cambium layers in the fleshy taproot of sugar beet, within which a large number of meristem cells possess the potential to regenerate shoots [36]. Shoot regeneration from adventitious roots in sugar beet could potentially be accomplished in the future through the exogenous addition of phytohormone and phytohormone transport or synthesis inhibitors.

## 4. Materials and Methods

### 4.1. Plant Materials and Cultivation

KWS9147 (KWS SAAT SE, Einbeck, Germany), a commercial variety, was selected for this study. Pelleted seeds were sowing in pots (12 cm × 12 cm × 10 cm) that contained black soil. Seedlings were grown for 10 days at 24 ± 2 °C, 400 µmol m^−2^ s^−1^ light intensity, and 16 h:8 h light/dark.

### 4.2. Induction of Adventitious Roots

After 10 days of growth, seedlings containing two expanded cotyledons were harvested for the induction of adventitious roots (Figure S1). The roots and a portion of the hypocotyl were excised approximately 0.5 to 0.8 cm below the cotyledons. For the induction of adventitious roots from leaves, fully expanded leaves in 14-day-old seedlings were cut at the base of the petiole. *A. rhizogenes* K599 was cultured in TY liquid medium (Yuanye, Shanghai, China) with shaking at 220 rpm and 28 °C overnight until the OD_600_ of the bacterial suspension reached 1.0. A 300 μL bacterial suspension was spread onto TY solid medium. Then, it was cultured at 28 °C until the medium was covered with a uniform layer of bacteria. Both bacterial suspensions and colonies were used for infection. There were three infection strategies, namely bacterial suspensions, colony, and two-step infection, which were designed to test the efficiency of *A. rhizogenes* K599 for induction of adventitious roots in shoots and leaves. For BSI, the hypocotyl wound was immersed in a medium containing *A. rhizogenes* K599 bacteria for 20 min. Colonies of *A. rhizogenes* K599 growing on solid medium were directly dipped 5 times onto the wound surface to act as BCI. The strategy of TSI was to successively implement the bacterial solution and colony infection. After infection, explants were directly transplanted into water-saturated vermiculite. Since the roots of the seedlings had been entirely removed, the vermiculite should hold sufficient water and the seedlings should be covered with plastic boxes or plastic wrap to guarantee a high humidity (about 70%) during the first week of cultivation. For each infection strategy, three biological replicates were established. Each replicate consisted of 12 explants for BSI and TSI and 38 explants for BCI. BCI was also employed to monitor the emergence of adventitious roots in infected shoots and leaves. Starting from 1 day after infection, the number of plants with adventitious roots was counted every 2 days. Three biological replicates were established, with each replicate consisting of 23 plants.

### 4.3. Detection of Genetic Transformation Efficiency

To examine the transformation efficiency of *A. rhizogenes*, eGFP with the *CaMV35S* and *MAS* promoter were constructed in using pCAMBIA1300 as the backbone, respectively (Figure S2). Plasmids were transformed into *A. rhizogenes* K599 by freeze–thaw method and then cultured in TY medium containing 50 µg/mL kanamycin. The fluorescence of eGFP in induced adventitious roots were photographed under a LUYOR-3415 light source (LUYOR, Shanghai, China). The transformation efficiency was calculated as the proportion of plants with positive adventitious roots to the total number of viable plants. Three biological replicates were set up. For shoots and leaves infected with plasmids containing *CaMV35S* and *MAS* promoters, each replicate contained 17, 30, 10, and 12 explants, respectively. To observe the development of adventitious roots and the distribution of eGFP expression after long-term growth, some transformed seedlings were transplanted into pots containing black soil for 3 months of growth. Fiji software (version 1.54p) was used for calculation of the relative fluorescence intensity by extracting the gray value of each pixel in the green channel of the region of interest [37,38].

### 4.4. The Validation of Transformation

*BvHDAC2* (*BVRB_006940*) were cloned from sugar beet cDNA and constructed to pCAMBIA1300-MAS::eGFP expression vector. The *A. rhizogenes* carrying pCAMBIA1300-MAS::BvHDAC2-eGFP and pCAMBIA1300-MAS::eGFP were used for infection. Three-month-old plants without infection were also set as the control. Roots with eGFP fluorescent from 3-month-old infected plants were harvested for total RNA and protein extraction.

The RNA extraction and cDNA synthesis were conducted as in our pervious study [39]. Briefly, 0.2 g roots were ground to powder in liquid nitrogen, and the total RNA was extracted using an RNA-easy isolation reagent (Vazyme, Nanjing, China) according to the instructions. RNA quality and concentration were determined using a NanoDrop 2000c (Thermo Fisher Scientific, Waltham, MA, USA). The cDNA was synthesized using a PrimeScript RT Reagent Kit (Takara, Dalian, China), according to the manufacturer’s instructions. A pair of *HPT* primers (Table S1) were also designed and utilized for PCR amplification. The PCR products were analyzed via electrophoresis (Liuyi, Beijing, China) on 1% agarose gel.

Quantitative real-time PCR (qRT-PCR) was carried out using the Mx3000P real-time PCR system (Agilent, La Jolla, CA, USA) with three biological replicates in TB Green premix Ex Taq (Takara, Dalian, China), following previous experimental protocols [39]. The primers for *BvHDAC2* were designed using Primer-BLAST, and *BvGAPDH* was used as the reference gene (Table S1). The relative levels of gene expression were calculated using the 2^−ΔΔCT^ method.

For Western blot, the total protein of the root was extracted using a plant total protein extraction kit (Sangon, Shanghai, China). Equal amounts of total proteins were then separated on a 12.5% SDS-polyacrylamide gel at 120 V for 90 min. Then, the proteins migrated from the gel onto a 0.2 μm polyvinylidene fluoride (PVDF) membrane (Merck, Darmstadt, Germany) in an electric field of 1.2 mA/cm^2^ for 40 min via a semi-dry transfer system (Liuyi, Beijing, China). The transfer buffer consisted of 25 mM Tris, 190 mM glycine, and 20% methanol. A 5% (*w*/*v*) reconstituted skimmed milk solution was used to block the membrane for 2 h. The membrane was incubated with anti-actin (D110007, Sangon) and anti-GFP (D19104, Sangon) antibodies at a 1:1000 dilution overnight, respectively. After being washed with a pH 7.6 20 mM Tris-HCl buffer containing 137 mM NaCl and 0.05% Tween 20, the proteins recognized by the primary antibody were revealed with a goat anti-mouse or anti-rabbit HRP-conjugated IgG at a 1:5000 dilution. The target proteins were visualized using SuperPico ECL master mix (Vazyme, Nanjing, China). The chemiluminescent signals were captured using a multifunctional imaging analysis system (Baygene, Beijing, China) in auto-exposure mode with an exposure time of 89 ms.

### 4.5. Statistical Analysis

In this study, SPSS software (version 25) was used for statistical analysis. The Duncan test was selected subsequent to a one-way ANOVA. The sample size and replication are reported in the figure legends.

## 5. Conclusions

In this study, a convenient and efficient *A. rhizogenes*-mediated transformation method for generation of composite sugar beet is established. It an especially optimal protocol for studying taproot development, sugar accumulation, and resistance to root diseases, which was closely related to sugar beet yield and quality. Additionally, our results supported the idea that the *MAS* promoter is more suitable for genetic transformation in sugar beet than the *CaMV35S* promoter, due to its higher transformation efficiency. We believe that this study will facilitate the development of molecular function research and molecular breeding in sugar beet.

## Data Availability

Data are contained within the article and Supplementary Materials.

## Supplementary Materials

The following supporting information can be downloaded at https://www.mdpi.com/article/10.3390/plants14172747/s1: Table S1: The primers of selected genes for PCR and qRT-PCR assay; Figure S1. The process of the Agrobacterium rhizogenes-mediated transformation; Figure S2. Schematic diagram of the eGFP expression plasmid used in this study; Figure S3. Comparison of eGFP expression in adventitious roots induced 21 days after infection by A. rhizogenes K599 with eGFP expression plasmid and without eGFP expression plasmid (CK). Scale bars = 1 cm. The MAS promoter is employed in the eGFP expression plasmid; Figure S4. Comparison of eGFP expression in adventitious roots induced 21 days after infection by A. rhizogenes K599 with eGFP expression plasmid K599 and without eGFP expression plasmid (CK). Scale bars = 1 cm. The MAS promoter is employed in the eGFP expression plasmid.

## Abbreviations

The following abbreviations are used in this manuscript:

qRT-PCR	quantitative real-time polymerase chain reaction
CDB	cut–dip–budding delivery system
eGFP	enhanced green fluorescent protein
CaMV35S	cauliflower mosaic virus 35S
MAS	mannopine synthase
BSI	bacterial suspensions infection
BCI	bacterial colony infection
TSI	two-step infection
HPT	hygromycin resistance
