# Supplementary material for: Agrobacterium rhizogenes-Mediated Transformation for Generation of Composite Sugar Beet with Transgenic Adventitious Roots
Source: Plants (Basel). 2025 Sep 2;14(17):2747. doi: 10.3390/plants14172747 (PMC12430162; doi:10.3390/plants14172747)
Supplement: Supplementary file 1 [file plants-14-02747-s001.zip › plants-3722403-supplementary/Supplementary Figure.pdf]

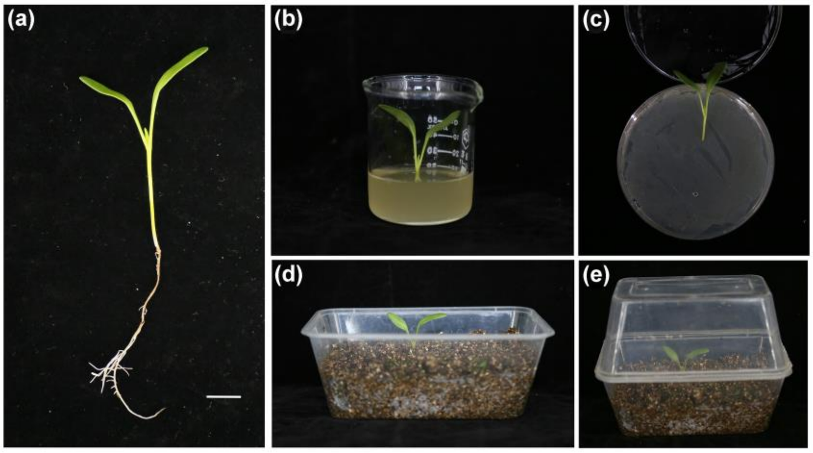

Figure S1. The process of the *Agrobacterium rhizogenes*-mediated transformation. (a) Morphology of 10-day-old seedlings; (b) The immersion of hypocotyl wound into the bacterial suspensions; (c) The hypocotyl wound was coated with *A. rhizogenes* K599 from the surface of the plate; (d) Infected seedlings were inserted into vermiculite cultures; (e) Inoculated seedlings were covered with a plastic cap. Scale bars = 1 cm.

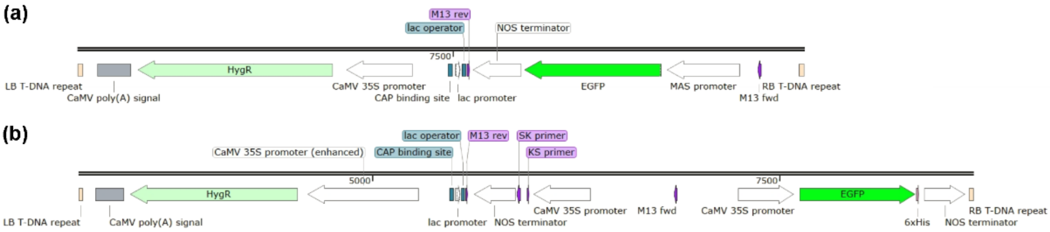

Figure S2. Schematic diagram of the eGFP expression plasmid used in this study. (a) eGFP with MAS promoter; (b) eGFP with *CaMV35S* promoter

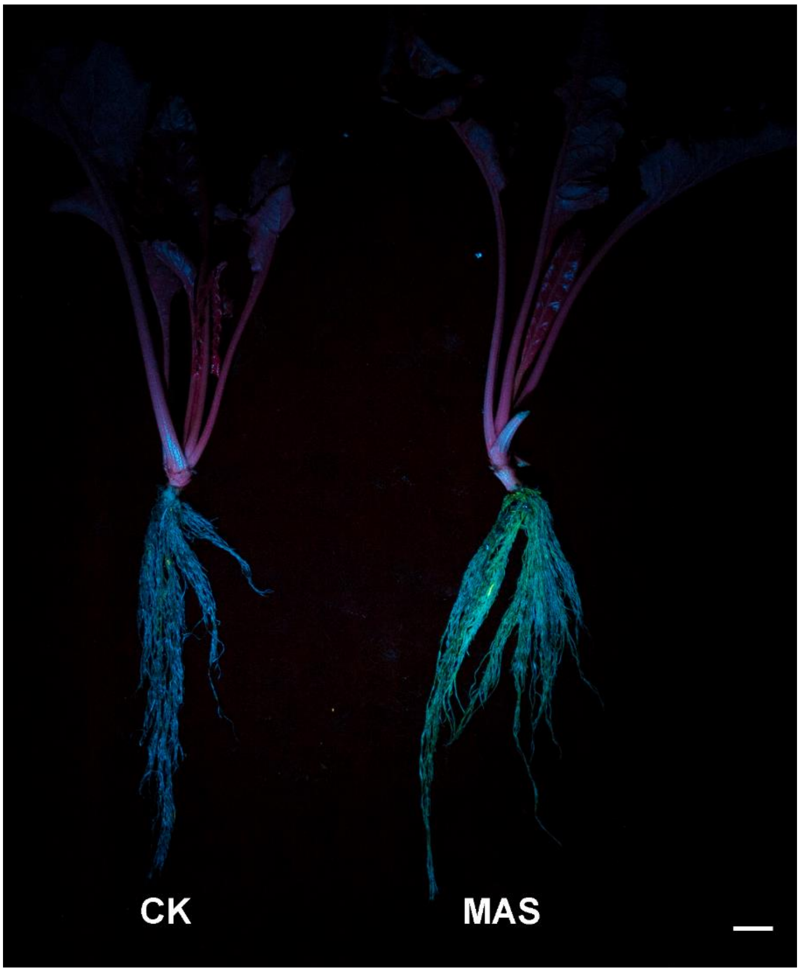

Figure S3. Comparison of eGFP expression in adventitious roots induced 21 days after infection by *A. rhizogenes* K599 with eGFP expression plasmid and without eGFP expression plasmid (CK). Scale bars = 1 cm. The MAS promoter is employed in the eGFP expression plasmid.

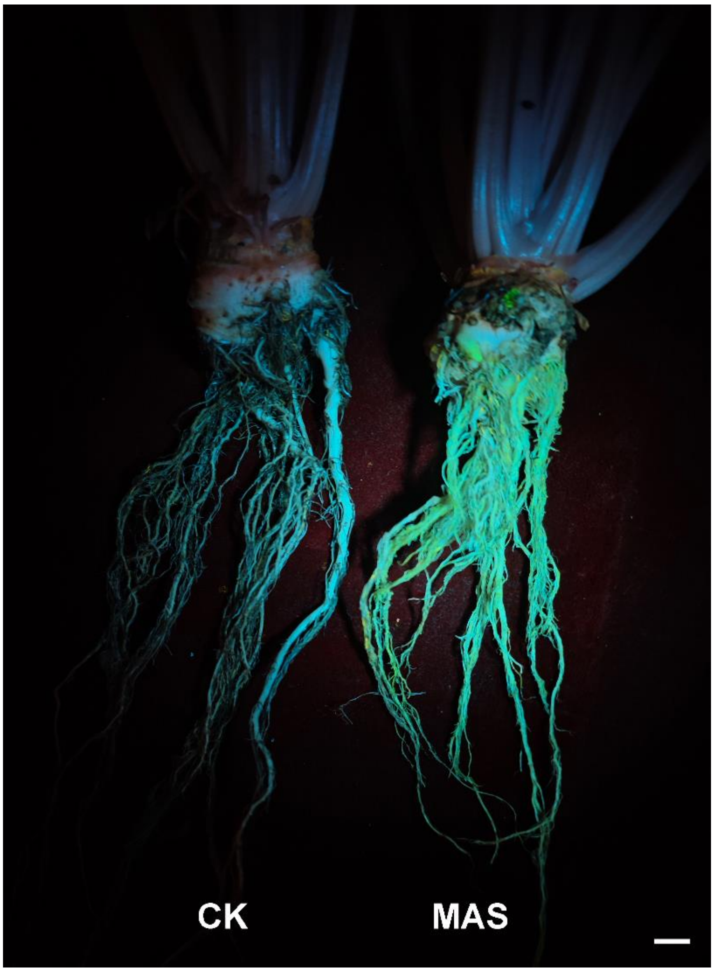

Figure S4. Comparison of eGFP expression in adventitious roots induced 21 days after infection by *A. rhizogenes* K599 with eGFP expression plasmid K599 and without eGFP expression plasmid (CK). Scale bars = 1 cm. The MAS promoter is employed in the eGFP expression plasmid.
