# Supplementary figures and images for: Agrobacterium rhizogenes-Mediated Transformation for Generation of Composite Sugar Beet with Transgenic Adventitious Roots
Source: Plants (Basel). 2025 Sep 2;14(17):2747. doi: 10.3390/plants14172747 (PMC12430162; doi:10.3390/plants14172747)

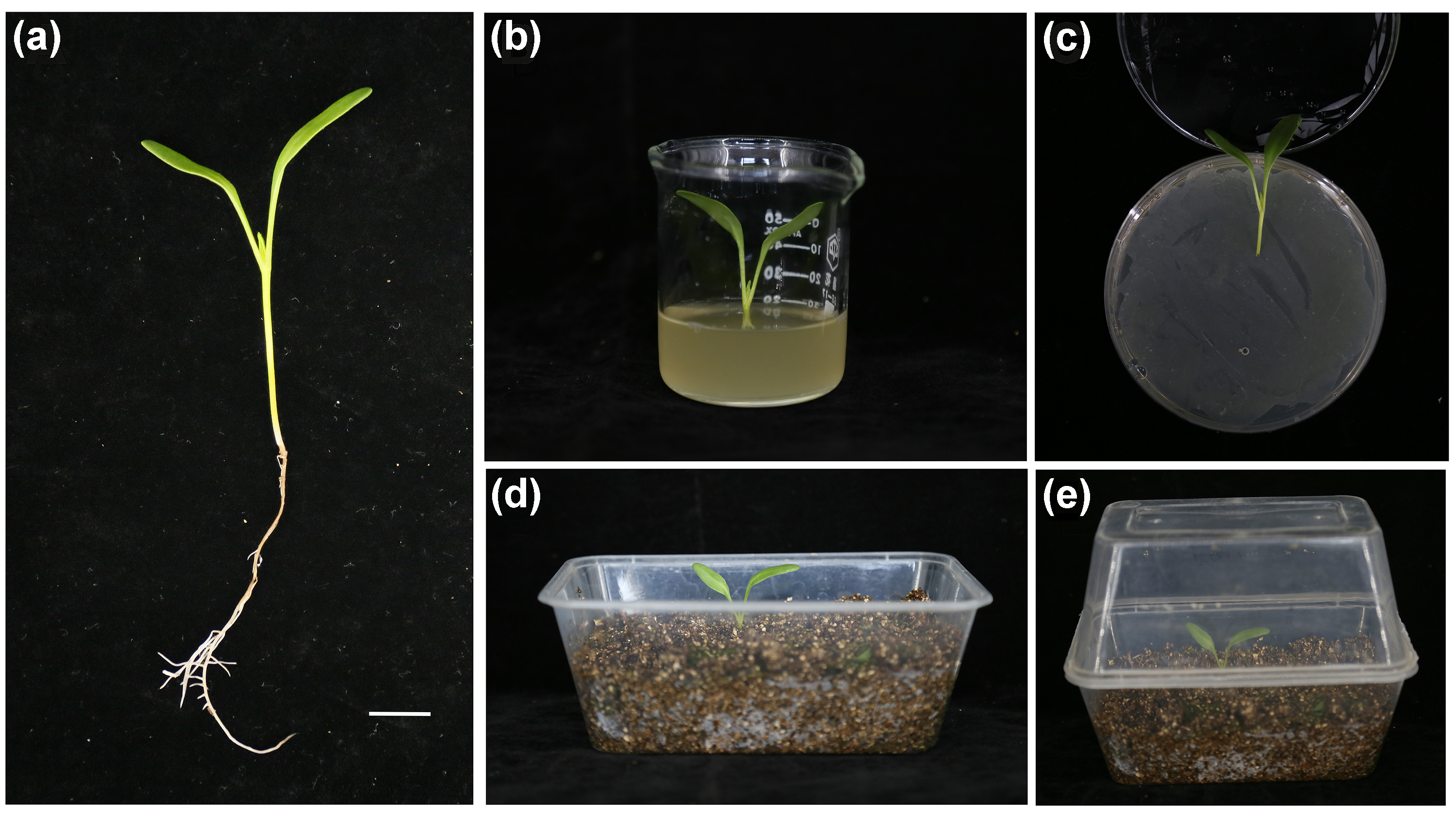

Supplement: Supplementary file 1 [file plants-14-02747-s001.zip › plants-3722403-supplementary/Figure S1.tif]

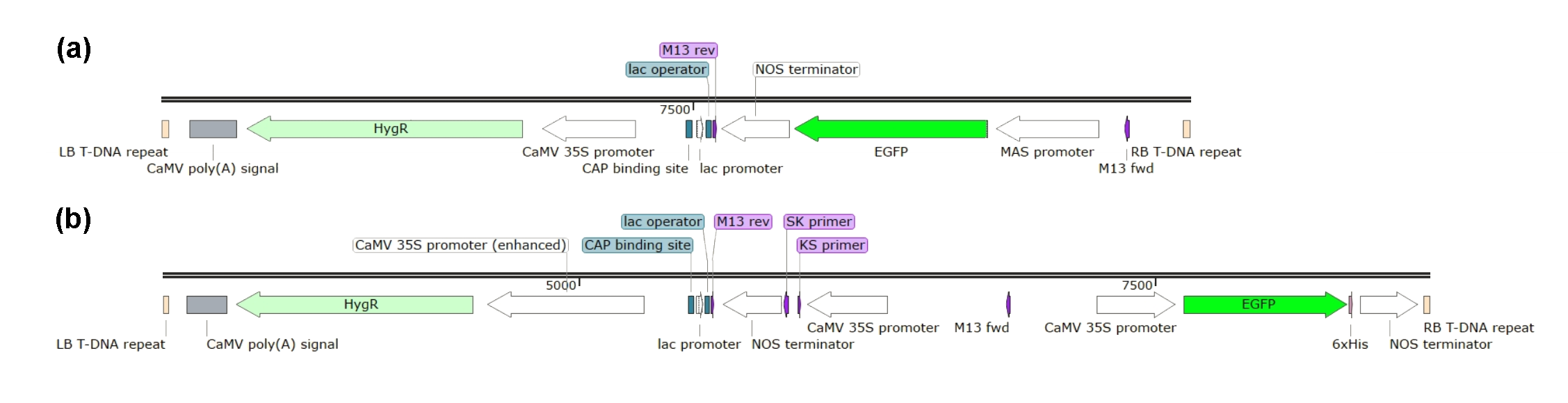

Supplement: Supplementary file 1 [file plants-14-02747-s001.zip › plants-3722403-supplementary/Figure S2.tif]

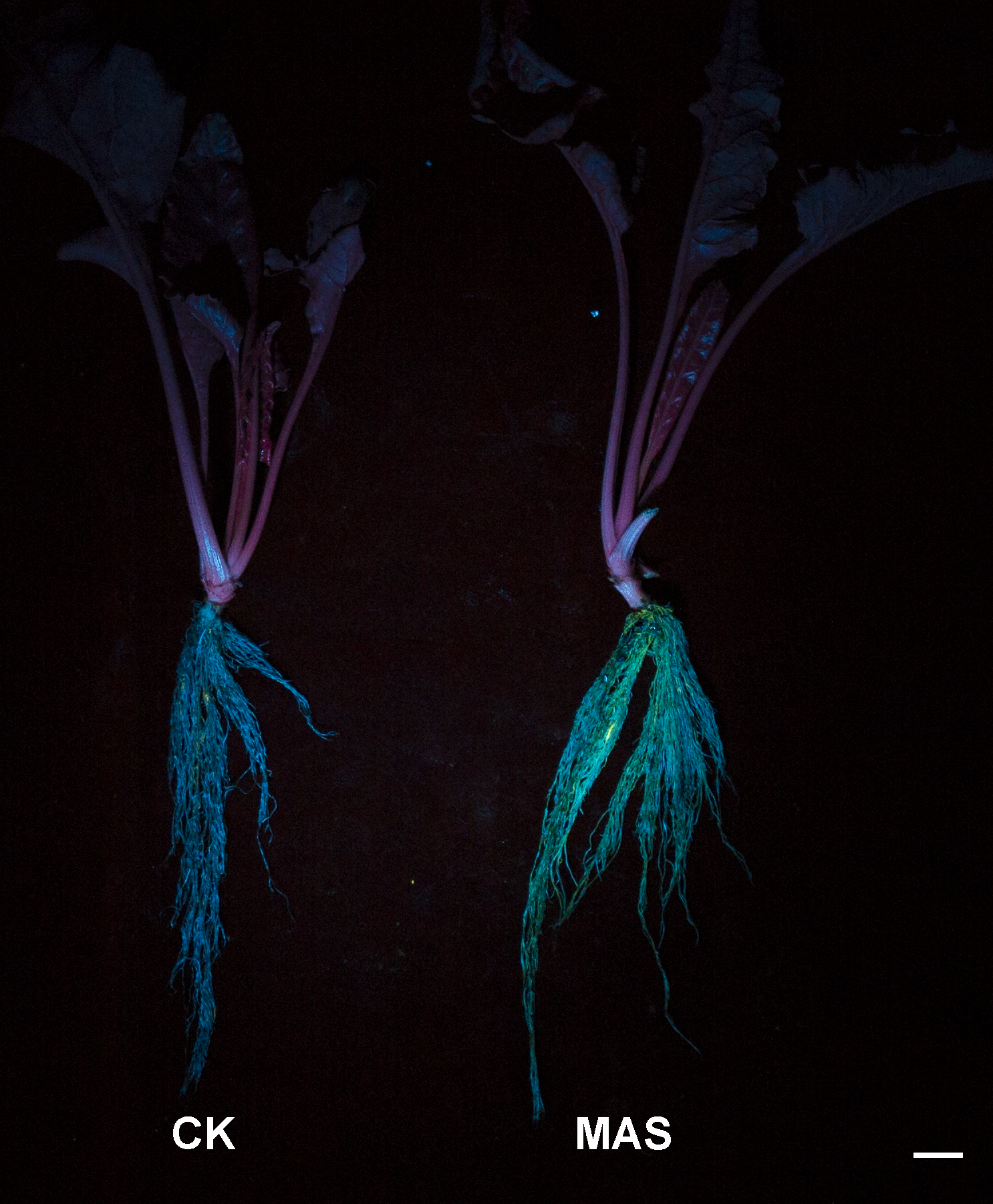

Supplement: Supplementary file 1 [file plants-14-02747-s001.zip › plants-3722403-supplementary/Figure S3.tif]

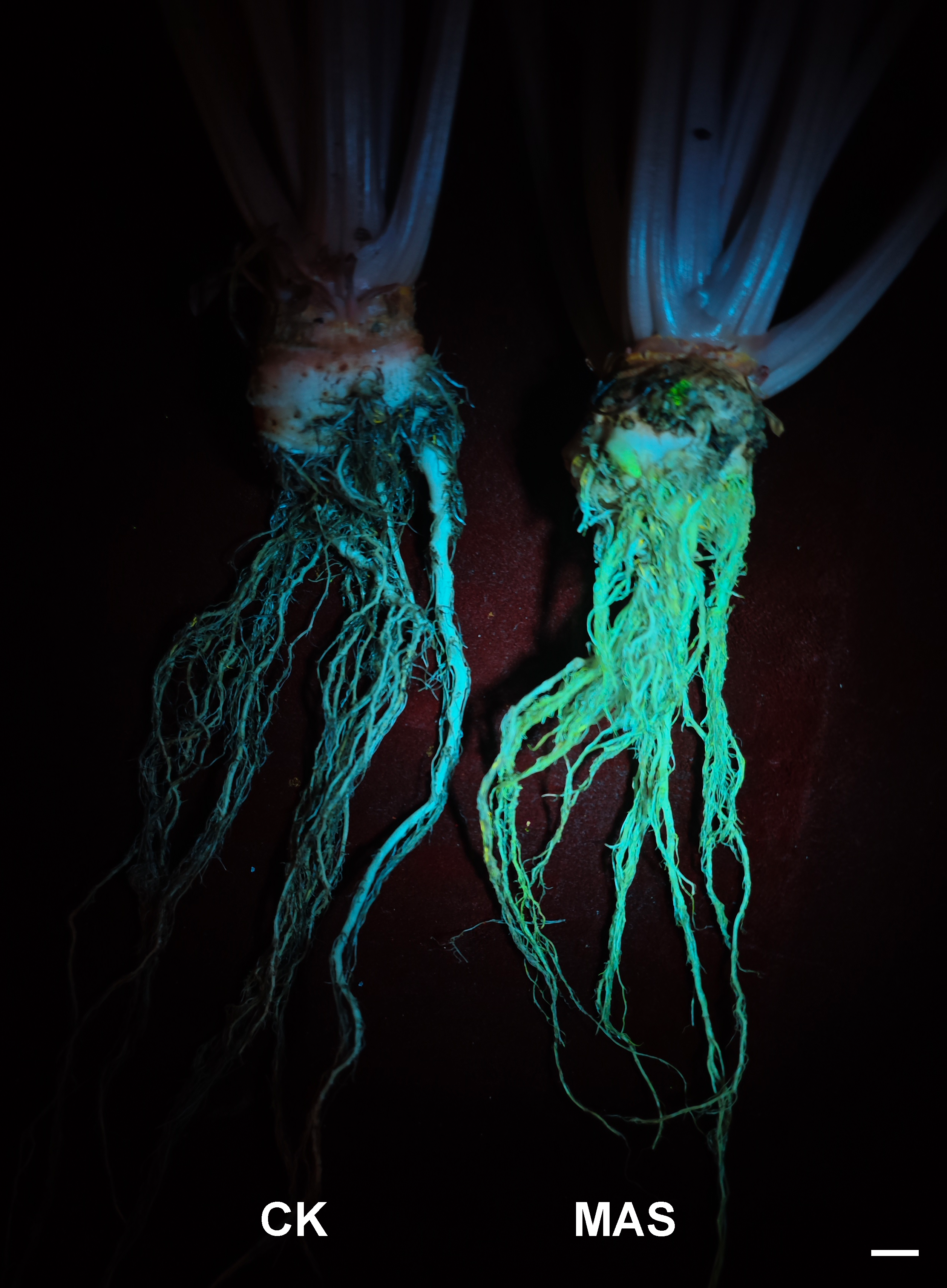

Supplement: Supplementary file 1 [file plants-14-02747-s001.zip › plants-3722403-supplementary/Figure S4.tif]
